# Photobiomodulation effects on neuronal transdifferentiation of immortalized adipose-derived mesenchymal stem cells

**DOI:** 10.1007/s10103-024-04172-2

**Published:** 2024-10-11

**Authors:** Heidi Abrahamse, Anine Crous

**Affiliations:** Laser Research Centre, Faculty of Health Sciences, University of Johannesburg, PO Box 17011, Johannesburg, 2028 South Africa

**Keywords:** Photobiomodulation, Near-infrared irradiation, Green light, Adipose-derived mesenchymal stem cells, Transdifferentiation, Neurogenesis

## Abstract

Adipose-derived mesenchymal stem cells (ADMSCs) possess the ability to transform into various cell types, including neurons. It has been proposed that the optimization of this transformation can be achieved by using photobiomodulation (PBM). The objective of this laboratory-based investigation was to induce the transformation of immortalized ADMSCs (iADMSCs) into neurons with chemical triggers and then evaluate the supportive effects of PBM at two different wavelengths, 525 nm and 825 nm, each administered at a dose of 5 J/cm^2^, as well as the combined application of these wavelengths. The results revealed that the treated cells retained their stem cell characteristics, although the cells exposed to the green laser exhibited a reduction in the CD44 marker. Furthermore, early, and late neuronal markers were identified using flow cytometry analysis. The biochemical analysis included the assessment of cell morphology, viability, cell proliferation, potential cytotoxicity, and the generation of reactive oxygen species (ROS). The findings of this study indicate that PBM does not harm the differentiation process and may even enhance it, but it necessitates a longer incubation period in the induction medium. These research findings contribute to the validation of stem cell technology for potential applications in in vivo, pre-clinical, and clinical research environments.

## Introduction

Adipose-derived mesenchymal stem cells (ADMSCs) can differentiate into various phenotypes, including neurons [1]. They are easy to harvest through surgery that is not very invasive and abundantly available [2]. The transdifferentiation process can be initiated through chemical transducers and biological growth factors [2–4]. Through these inducers, the in vitro differentiation potential of the ADMSCs are vastly increased and cells can be transdifferentiated into multiple lineages such as chondrocytes, neural cells, adipocytes, or osteocytes [2]. Previous research has shown that it is possible to differentiate ADMSCs into neuronal lineages [5, 6]. These studies made use of inducers that included basic fibroblast factor (bFGF), forskolin, and 3-isobutyl-1-methylxanthine (IBMX). There is a suggestion that ADMSCs may have the potential to be applied in a clinical context to tackle neurodegenerative disorders like Alzheimer's disease or amyotrophic lateral sclerosis (ALS) [7, 8].

Photobiomodulation (PBM) is the application of laser light to tissue that can increase or decrease cellular metabolism. The effect of laser light on tissue is dependent on the laser parameters applied [9]. These parameters include the energy applied or fluency in J/cm^2^ and wavelengths that range from 400–1100 nm covering the visible and near-infrared spectrum (NIR) [10–13]. Fluency is a dose dependent value which has been determined to be ineffective in upregulating ADMSC proliferation at 0.5—2 J/cm^2^ and effective at 3 – 5 J/cm^2^ [4, 14]. In terms of its mechanism, PBM activates chromophores located in the mitochondria, leading to the stimulation of the electron transport chain, which produces an increased quantity of adenosine triphosphate (ATP). This, in turn, enhances the mitochondrial membrane potential (MMP) [15, 16]. Research has shown that wavelengths on the visible spectrum, such as 450 nm and 580 nm results in an increase in ADMSC differentiation into osteoblasts [17, 18]. Green (G) laser light has also been applied to facilitate the stimulation of transcription factors that play a role in cellular differentiation [19]. Furthermore, it has also been seen that wavelengths on the visible spectrum, such as 451 nm and 540 nm do not necessarily increase the proliferation of cells, while wavelengths on the red and NIR side of the spectrum of light, such as 660 nm and 810 nm results in the upregulation of ADMSCs [10]. NIR laser light also has a significant effect on the migration of ADMSCs which is necessary for the homing of cells to the site of injury [20]. The current study considered the effect of 525 nm G, 825 nm NIR laser light at 5 J/cm^2^ and their combination (NIR-G) for a total of 10 J/cm^2^ on the transdifferentiation of ADMSCs into neuronal lineages. The effect of these laser parameters on cellular morphology, viability, proliferation, cytotoxicity, ROS, and neuronal marker expression was considered.

## Methodology

### Cell culture

hTERT ASC52telo (ATCC® SCRC-4000™) cells were cultured in Dulbecco's Modified Eagle Media (DMEM) (Sigma-Aldrich, D5796) with an addition of 10% fetal bovine serum (FBS Superior) (Biochrom, S0615), and the culture medium was supplemented with 1% antibiotics, comprising 0.5% Penicillin–Streptomycin (Sigma-Aldrich, P4333) and 0.5% Amphotericin B solution (Sigma-Aldrich, A2942). The cells were grown in a controlled environment at 37 °C with 5% CO_2_ and 85% humidity inside a Heracell™ 150i CO_2_ Incubator (Thermo Scientific™, 51026280). They were cultured in cell culture flasks from Corning® (Sigma, CLS430639/ CLS430641/ CLS431080). Once the cells reached full confluence, they were transitioned to a specialized neuronal differentiation medium containing the specified components: Indomethacin [200 µM] (I7378-10G, Merck/SIGMA), Insulin [5 µg/ml] (I9278-5ML, Merck/SIGMA), and 3-Isobutyl-1-methylxanthine (IBMX) [0.5 mM] (I5879-100MG, Merck/SIGMA).

### Photobiomodulation

After the cells were cultured, approximately 100,000 cells were placed in 35 mm diameter petri dishes (Corning, 430165) with complete medium and left to incubate for 24 h before the irradiation process. The attached ADMSCs were subjected to two types of lasers: a NIR 825 nm Diode Laser (National Laser Centre of South Africa, SN 070900108) equipped with a 1000 mA LaserSource (ArroyoInstruments, 4210) and a G 525 nm Diode Laser (National Laser Centre of South Africa, EN 60825–1:2007) with a power source of 100–240 VAC and 47–63 Hz at 5A (OptoElectronics Tech.CO.,LTD). The laser's output power in milliwatts (mW) was measured using a FieldMate Laser Power Meter (Coherent, 1098297). The irradiation time was determined based on the energy output, using a High-Sensitivity Thermopile Sensor PM3 (Coherent, 1098336). The laser spot size was sufficient to cover the entire cell monolayer. Specific laser parameters are detailed in Table 1 for reference.

**Table 1:** Laser parameters

	Near infra-red (NIR)	Green (G)
Wavelength (nm)	825	525
Type	Diode	Diode
Emmision	CW	CW
Power (mW)	100	574
Power density (mW/cm^2^)	10.394	59.66
Fluence (J/cm^2^)	5	5
Time of irradiation (s)	8 min 1 s	1 min 23 s
Spot size (cm^2^)	9.62	9.62

The cell cultures were segregated into three distinct experimental groups: 1) cells subjected to NIR irradiation at 825 nm with a dosage of 5 J/cm^2^, 2) cells subjected to G irradiation at 525 nm with a dosage of 5 J/cm^2^, and 3) cells treated with NIR at 825 nm followed immediately by G at 525 nm, both at a fluency of 5 J/cm^2^. Cells that were not exposed to laser treatment (0 J/cm^2^) were included as control samples. The duration of exposure for each laser wavelength was determined using the following formula:

$$mW/{ cm}^{2}=\frac{mW}{\pi {r}^{2}}$$

$$W/ {cm}^{2}=\frac{mW /{ cm}^{2}}{1000}$$

$$Time (\text{seconds})=\frac{ J/{cm}^{2}}{W/{cm}^{2}}$$

### Characterization of markers

#### Flow cytometry

Secondary antibody conjugation was used to label cultivated hTERT ASC52Telo (ATCC® SCRC-4000™) cells with a fluorescent marker that was subsequently observed through flow cytometry (BD 468 Biosciences, BD ACCURI C6 PLUS). According to the ATCC® guidelines, the CD44 marker was tested for. Cultivated cells were cultured with complete media in sterilized 35 mm diameter petri dishes (Corning, 430165) with a seeding density of 1 × 10^5^. An incubation period of 24 h was used to adhere cells to the petri dish. Cells were washed three times with a cold washing solution (Azide/PBS/BSA: containing 0.01% w/v Sodium azide from Sigma-Aldrich, PBS, and 0.1% w/v BSA from Sigma-Aldrich). Afterward, the ADMSCs were chilled and treated with a blocking solution for 30 min (BSA/PBS: consisting of 10% w/v BSA in PBS). The cells were washed three times with the cold washing solution again. Next, the cells were exposed to the primary antibody, anti-CD44 (ThermoFisher, MA110225), which was diluted in a working solution (Azide/PBS/BSA/FBS: containing 0.01% w/v sodium azide from Sigma-Aldrich, PBS, 0.1% w/v BSA, and 2% FBS from Biochrom) for 30 min on ice. Subsequently, the cells were washed three times with the washing solution and then incubated with the necessary secondary fluorescent antibody, Cy5 goat anti-mouse (NovusBio, NB7602), for 30 min in the dark on ice. The washing step was repeated. Following this, the cells were fixed with 10% paraformaldehyde (P6148, Sigma-Aldrich) for 10 min. After fixation, the cells were washed three times with PBS and finally resuspended in PBS. The cells were promptly analyzed using a flow cytometer (Fl-4, Cy5 filter) from BD Biosciences (BD ACCURI C6 PLUS).

### Biochemical analysis

#### Morphology: inverted light microscopy

Morphological changes were examined and analyzed at 24 h, 48 h, and 7 days after laser treatment using inverted light microscopy (OLYMPUS CKX41). These observations were captured with a digital camera attached to the microscope (OLYMPUS, SC30), and the images were processed using the cellSens software.

#### Cell viability: trypan blue exclusion assay

A color exclusion assay was conducted to assess cell viability using the Trypan Blue Staining method (0.4%) from Invitrogen (T10282). The Countess Automated Cell Counter (ThermoFischer, AMQAX1000) was employed to visualize the cells and, subsequently, calculate the percentage of viable cells.

#### Proliferation: ATP luminescence assay

Cell proliferation was assessed using the ATP luminescence assay known as CellTiter-Glo® 2.0 (Promega, G9241). The luminescent intensity was quantified by the VICTOR3 Multilabel Plate Counter (PerkinElmer, HH3522019094), which measured the relative light units (RLUs).

#### Lactate dehydrogenase

The LDH cytotoxicity assay was employed to quantify the release of lactate dehydrogenase (LDH) using the CytoTox 96® non-radioactive cytotoxicity assay. This assay assesses the LDH released due to cell membrane damage by measuring the amount of LDH generated during the conversion of lactate into pyruvate. It is a colorimetric assay that produces a red by-product, and its reactivity depends on NADH. The measurement was carried out at a wavelength of 490 nm using a spectrophotometer.

#### Reactive oxygen species (ROS) detection – spectrophotometry

To measure ROS production and to determine whether cellular damage occurred due to stimulated ROS generation, the Image-IT® LIVE Green Reactive Oxygen Species Detection Kit (Life Technology I36007) was used. ROS, along with free radicals, are generated during cellular metabolism as part of redox reactions when cells are stimulated externally. When calcein and fluorescein levels are low, the presence of ROS is indicated. When the acetate groups of 2’, 7’-di-chlorofluorescein (DCF) and calcein are removed through intracellular esterases, oxidation occurs, and fluorescence is observed. Using this method, ROS was observed through spectroscopic means (VictorNivo).

### Statistical analysis

Biochemical assays were carried out in triplicate (*n* = 3). Spectrophotometric investigations included the incorporation of a blank sample generated from the collected data. To analyze the data, statistical tests were employed: the Student's t-test for comparing two groups and one-way ANOVA for comparing all groups. These analyses were performed using the SigmaPlot program version 12. Morphological data was quantitatively assessed using ImageJ, which is a publicly accessible Java-based image processing system developed by the National Institute of Health in Bethesda, MD, USA. The data is presented as the mean ± standard error (SE). Statistical significance is indicated in tables and graphs as follows: *P* < 0.05 (*), *P* < 0.01 (**), and *P* < 0.001 (***), and the standard error is visually represented using error bars.

## Results

### Characterization of markers

**Fig. 1:**
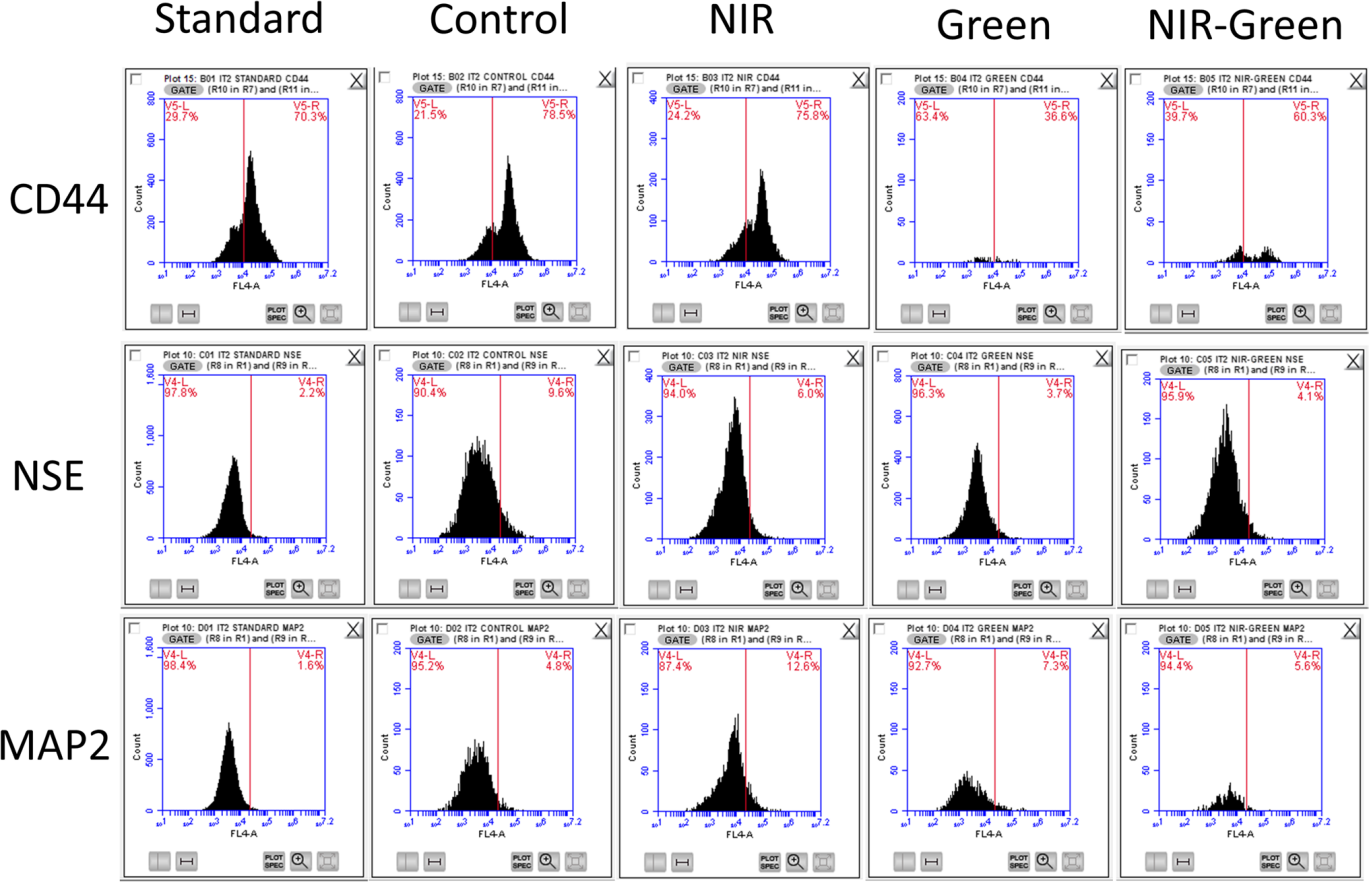
Flow cytometry analysis of CD44 marker, early neuronal marker Neuronal Specific Enolase (NSE), and late neuronal marker Microtubule-associated protein 2 (MAP2) was conducted. The results show the presence of early (NSE) and late (MAP2) neuronal markers 7 days after photobiomodulation (PBM) treatment, although the percentages were relatively low. Previous studies indicate that early neuronal markers like NSE typically appear after 14 days of incubation with differentiation media, with no detection of late markers or the generation of functional neuronal cells. Notably, green (G) laser light was the most effective in reducing stem-like characteristics by almost half compared to other groups, highlighting its efficacy in differentiating induced adipose-derived mesenchymal stem cells (iADMSCs)

ADMSCs commercially obtained from ATCC were characterized using the suggested CD44 marker. This determined whether cell’s-maintained stem-ness or lost stem-ness as the cells transdifferentiated. Flow cytometry was employed to analyze the CD44 marker, as well as the early neuronal marker Neuronal Specific Enolase (NSE) and the late neuronal marker Microtubule-associated protein 2 (MAP2). The flow cytometry analysis detected the presence of both early (NSE) and late (MAP2) neuronal markers 7 days after the PBM treatment. However, the percentages of these markers were found to be very small. A control sample was run in flow cytometry analysis to determine the population of interest through cell gating. Fluorescence was detected with a FL-4 Cy5 filter. Flow cytometry characterization results of CD44 revealed that the marker was maintained (Fig. 1). It is worth noting that flow cytometry results indicated that ADMSCs treated with G 525 nm PBM had the lowest percentage of CD markers compared to that of the other wavelengths, standard and control.

### Biochemical analysis

#### Morphology

**Fig. 2:**
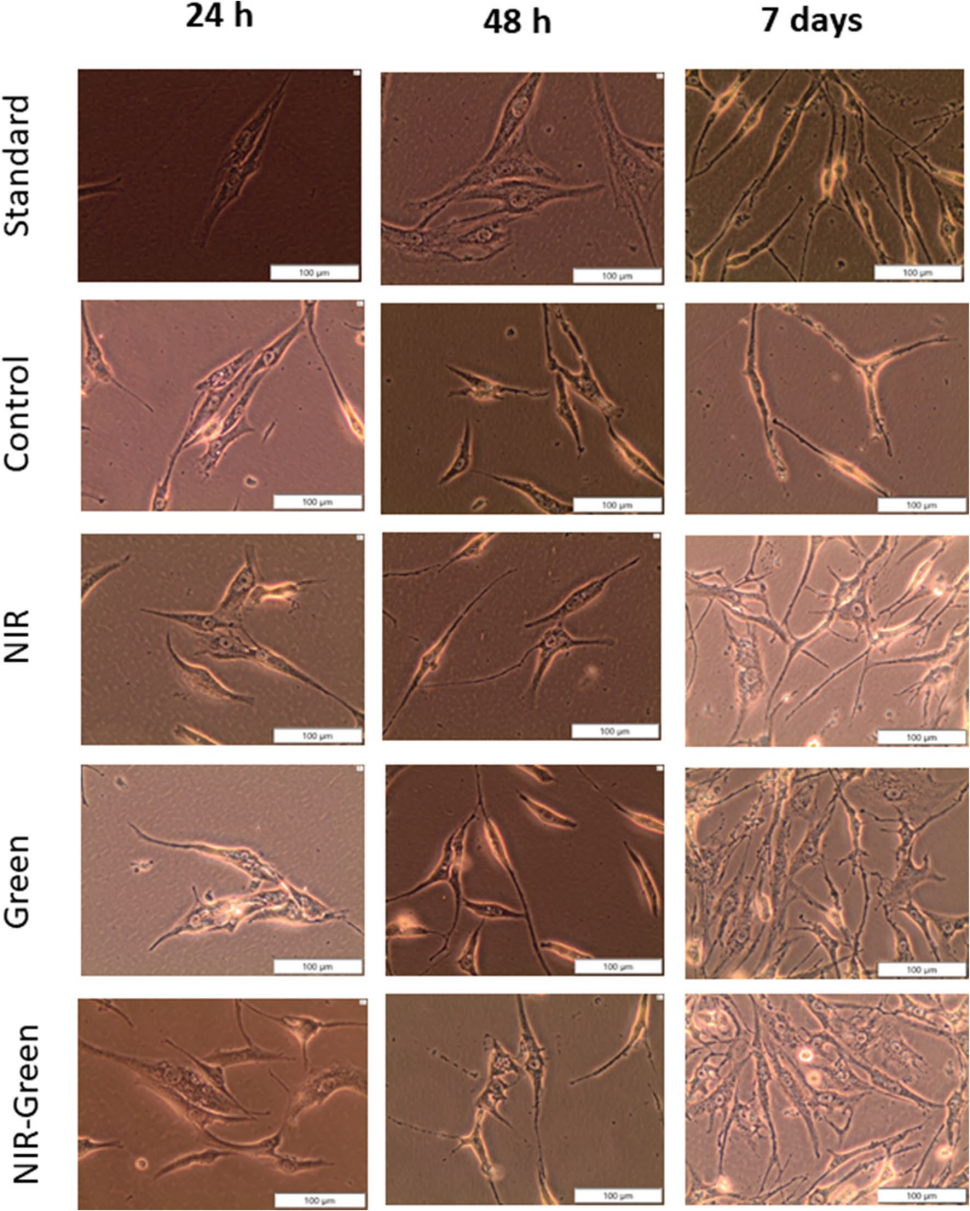
Morphology of iAMDSCs post 24 h, 48 h, and 7 days after PBM treatment. Morphological observations indicated distinct differences between the control group, characterized by the typical smooth cell morphology of healthy ADMSCs, and the experimental groups of ADMSCs exposed to laser irradiation at varying wavelengths and a fluence of 5 J/cm^2^. Notably, the group treated with 525 nm green laser light and NIR-G laser light displayed the highest cell density seven days after photobiomodulation (PBM) exposure

Observations of morphology showed changes in treated groups from that of the control (Fig. 2). The control group exhibited the usual smooth cell morphology that is characteristic of healthy ADMSCs. In contrast, the experimental groups of ADMSCs that underwent laser irradiation at varying wavelengths (525 nm and 825 nm) and a fluence of 5 J/cm^2^ displayed noticeable changes in their morphology compared to the control group. Among these experimental groups, the one treated with 525 nm green laser light and NIR-G laser light exhibited the highest cell density 7 days after the PBM exposure.

#### Cell viability and proliferation

**Fig. 3:**
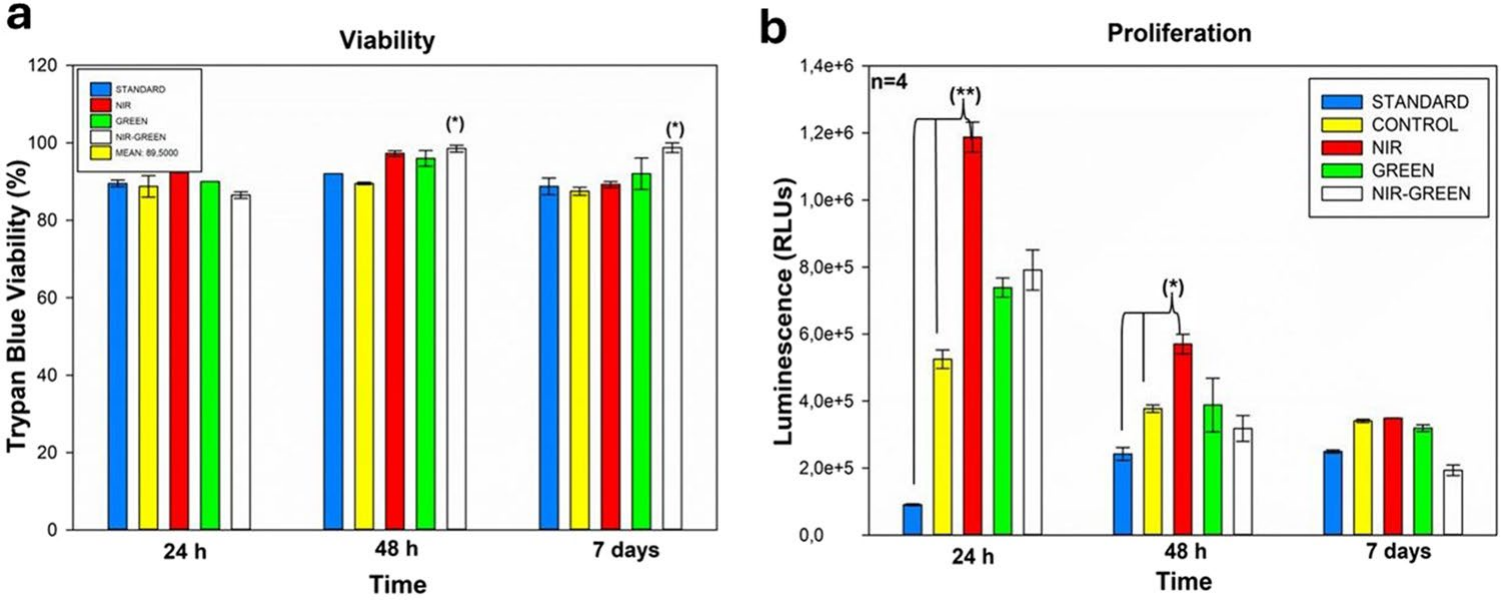
Trypan blue viability assay (**a**). Biochemical analysis demonstrated a consistent percentage of viable cells at 24 h, 48 h, and 7 days following PBM exposure. Cellular proliferation (**b**) (ATP Luminescence) indicated a notable rise in ATP levels in the experimental group treated with NIR PBM at both 24 and 48 h after PBM exposure. However, 7 days after PBM exposure, there was no significant increase in proliferation, which might be attributed to the cells redirecting their energy towards differentiation rather than proliferation. *P* < 0.05 (*), *P* < 0.01 (**), and *P* < 0.001 (***)

The trypan blue dye exclusion assay was used to establish the effects of transdifferentiation media and PBM on cell viability (Fig. 3a). Cells with degrading membranes take up this dye, whereas cells with intake membranes, the viable cells, will not allow the dye to enter into the cells. Results indicated no significant effects on the percentage viability of cells whether exposed to transdifferentiation media or the NIR-G of transdifferentiation media and PBM compared to the standard.

The impact of transdifferentiation and PBM on the proliferation and metabolic activity of iADMSCs was examined using the ATP luminescence assay, as shown in Fig. 3b. This assay employs luciferase to produce a luminescent signal, which is proportional to the ATP level in the test sample. ATP concentration is directly associated with the rate of cell proliferation, suggesting an increase in mitochondrial activity and cellular growth. Cell proliferation was observed at three time points: 24 h, 48 h, and 7 days after laser treatment. The findings demonstrated that transdifferentiated cells treated with NIR PBM displayed a noteworthy increase in proliferation at both 24- and 48-h post-irradiation. However, there were no significant differences observed in proliferation 7 days after PBM irradiation.

#### Lactate dehydrogenase (LDH)

When the cell membrane is compromised, lactate dehydrogenase (LDH) is released into the cytosol. This property can be utilized to identify cytotoxicity within the cell. The NADH-dependent CytoTox 96® Non-Radioactive Cytotoxicity Assay (Promega, G1780) transforms tetrazolium salt into a red formazan, which can then be quantified using a spectrophotometer in a flat-bottom Corning® 96 Well Clear Polystyrene Microplate (Sigma, CLS3370). The concentration of formazan generated is directly proportional to the cytotoxicity of the cells, serving as an indicator of the extent of cell damage or cell death.

**Fig. 4:**
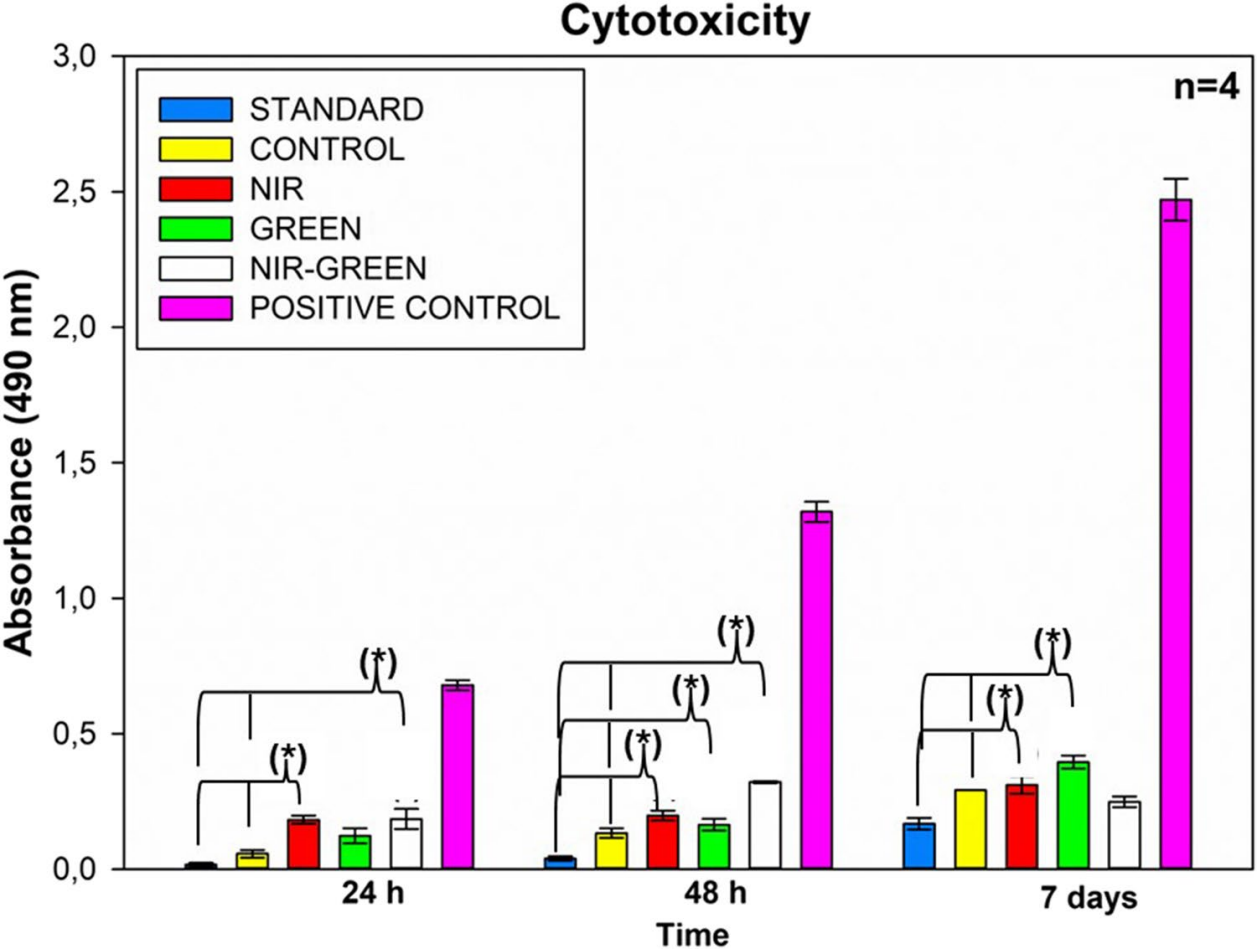
Lactate dehydrogenase (LDH) cytotoxicity assay. Following PBM treatment, there was an increase in LDH production at 24 h for the NIR and NIR-G wavelengths, with a significant rise in LDH concentration in all experimental groups at 48 h post-treatment. Additionally, NIR and G PBM led to a significant increase in LDH concentration 7 days after irradiation. However, these increases, while significant compared to the standard and control groups, did not reach levels indicative of severe harm when compared to the positive control, representing 100 percent toxicity and cell death. *P* < 0.05 (*), *P* < 0.01 (**), and *P* < 0.001 (***)

A notable rise in LDH levels was observed in both the PBM NIR and NIR-G treated groups 24 h after PBM exposure (Fig. 4). Similarly, a significant increase in LDH levels was noted in all experimental groups subjected to PBM at the 48-h mark post-exposure. Finally, at 7 days after irradiation, NIR and G laser light treatments displayed a significant increase in cytotoxicity. However, it's worth noting that when compared to the positive controls, this cytotoxicity is not indicative of apoptosis induction in the cells.

#### Reactive oxygen species (ROS)

**Fig. 5:**
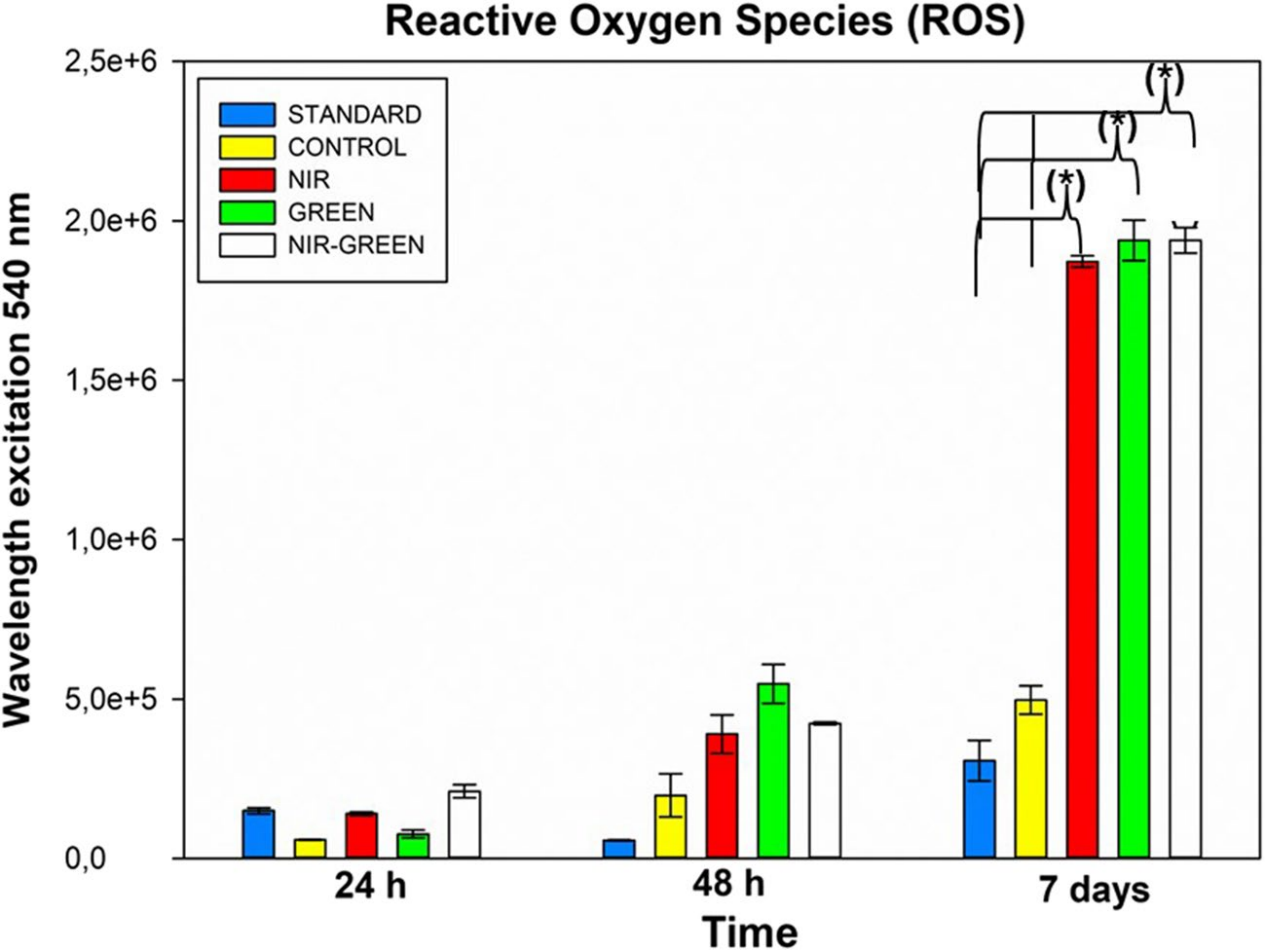
Reactive oxygen species (ROS) assay. Increased ROS activity was noted with NIR and concurrent PBM at 24 h, as well as for all PBM groups at 48 h, although statistical significance was not reached. However, a significant increase in ROS production was observed in all experimental groups compared to the standard and control following seven days of PBM exposure, indicating a potential role in guiding stem cell fate. Notably, this rise in ROS levels did not harm the cells, as evidenced by the viability results. *P* < 0.05 (*), *P* < 0.01 (**), and *P* < 0.001 (***)

Physiological increments of ROS generation is necessary for the maintaining of the activity of stem cells, however, ROS concentration above a certain threshold becomes cytotoxic. Thus, ROS expression was measured in the iADMSCs to determine the effects of transdifferentiation induction and PBM had on the intracellular production of ROS. A negligible increase in ROS production was seen in PBM treated groups compared to the standard 48 h post irradiation (Fig. 5). NIR, G, and the NIR-G wavelengths showed a significant increase in ROS production following 7 days after laser exposure with G having the highest concentration in ROS production. When compared to the above-mentioned proliferation, cytotoxicity, and viability studies, it should be noted that the increase in ROS concentration was not detrimental to the cells.

## Discussion

Multiple studies have explored the possibility of transdifferentiating ADMSCs into other cell types, such as neuronal or osteogenic cell types. It is well documented that adult tissues contain different stem cells that can be isolated. These stem cells can repair tissue damage regeneratively. Additionally, stem cells are not limited to specific lineages and show a remarkable plasticity in adapting to other lineages [21]. In this study, it was demonstrated that iADMSCs can transdifferentiate into cells that display neuronal markers and resemble the morphology of immature neurons. This study demonstrated that this process may be augmented with PBM [4].

The effect of PBM on ADMSCs has also been explored, although the concept of combining two wavelengths to achieve stronger proliferation and differentiation simultaneously is still novel. PBM has been shown to increase proliferation when NIR wavelengths are applied and increase differentiation into osteogenic lineages when G wavelengths are applied [19]. Many benefits exist for neuronal cells derived from ADMSCs to be clinically applied. ADMSCs are readily available in large amounts with small comprise to morbidity and overcomes the issue of isolating cells from the brain itself [22]. These cells can also be easily expanded in vitro and are renewable.

The transducers used in this study were insulin, IBMX, and indomethacin. Insulin was applied to upregulate maturation of differentiating neurons. IBMX was used to elevate intracellular adenosine monophosphate (AMP), which in turn acts as a neuronal stimulus. Indomethacin was used due to its ability to promote neuronal cell survival. Prior to transduction, the morphology displayed by iADMSCs are flat and spindle-like in structure. Following transduction, the cells should show neuronal-like structural changes. In this study, some morphological changes were seen in comparison to that of the standard. The treated cells showed a more rounded morphology to that of the standard.

Flow cytometry analysis showed a maintenance of CD44, indicating a maintaining of the iADMSC stem-ness. Flow cytometry analysis of the early neuronal markers, Neuronal Specific Enolase (NSE), and late neuronal marker, microtubule-associated protein 2 (MAP2), indicated that cells were transdifferentiating albeit in very small percentages. Previous studies show that early neuronal markers such as NSE can be seen after 14 days of incubation with differentiation media, no late markers were detected, and functional neuronal cells were not generated [23]. Green PBM was the most effective in reducing stem-ness by almost half compared to that of other groups, indicating it is an effective tool in differentiating iADMSCs. This augmentation of differentiation by G PBM has also been observed when differentiating ADMSCs into osteogenic lineages [4]. The authors of this paper would suggest that, although PBM does upregulate transdifferentiation in that late neuronal markers were observed after only 7 days, this may be further enhanced by a longer incubation period.

Biochemical analysis revealed that viability remained largely unaffected irrespective of the treatment introduced to the cells. This maintenance of viability indicates that PBM is not detrimental to the iADMSCs in vitro. These results are similar to studies by Mvula et al. who exposed ADMSCs to NIR PBM after which the viability maintained a high percentage [9]. Previous research has also recorded no significant effects on viability following G PBM exposure at wavelengths 405 nm [24] and 470 nm [25].Thus, the viability maintenance seen in the NIR-G PBM experimental groups remains insignificant in comparison to the standard.

Proliferation results indicated a steady but negligible decrease following PBM. Cells exposed to NIR PBM showed a significant increase in ATP proliferation 24 h and 48h post irradiation. The upregulation in proliferation following NIR exposure is caused by stimulation of the metabolism as observed by the increase in ATP production. The increase in proliferation seen following NIR PBM was also observed by various research groups [14, 26]. No significant increase in proliferation was noted in this study where induction media acted as a second variable instead the general trend in this study shows a steady decrease in proliferation. As the cells maintained their viability, it may be suggested that the steady decline in proliferation may be due to cells redirecting their energy for differential purposes. This correlates to a study by Cardozo et al. in which cells were treated with another type of transdifferentiation media, but not PBM, also indicated a decrease in proliferation [27], concluding that proliferation naturally decreases as differentiation increases. Similarly, this phenomenon can be explained by cell crowding or cell density-dependent inhibition of cell proliferation. This phenomenon is closely related to contact inhibition, where cells cease dividing when they encounter neighboring cells. As cell density increases, nutrient and oxygen availability can become limited, leading to decreased ATP production and a slowdown or cessation of cellular processes, including proliferation.

Cytotoxicity was assessed by measuring the concentration of LDH in the samples, and these results were also compared to a positive control. At 24 h after PBM treatment, both NIR and NIR-G groups displayed a notable increase in LDH concentration compared to the standard. Similarly, all three PBM experimental groups exhibited a significant increase in cytotoxicity when compared to the standard 48 h after PBM treatment. Finally, 7 days after PBM exposure, both NIR and G PBM led to a substantial increase in LDH concentration when compared to the control. However, when these significant increases were compared to the positive control, they were not substantial enough to induce apoptosis or indicate severe harm to the cells.

The standard group exhibited a slight increase in ROS concentration over the 7-day period, suggesting that ROS levels rise with the growth of the cell population. In contrast, the experimental groups treated with PBM displayed a noteworthy increase (*p* < 0.05) in ROS levels 7 days after exposure. This upregulation has been noted in other differentiation studies where ROS concentration physiologically increases to direct the fate of the stem cells [28]. Furthermore, when comparing these results to that of the viability and cytotoxicity results, it can be seen that this increase was not detrimental to the cells. Interestingly, elevated ROS levels are seen not only in neuronal development studies but also in various functions performed by neurons [29].

## Conclusion

In conclusion, the in vitro study aimed to differentiate iADMSCs into neuronal-like cells by culturing them in 35 mm well plates and exposing them to an induction medium containing IBMX, insulin, and indomethacin. Subsequently, the cells underwent treatment with NIR laser light at 825 nm and G laser light at 525 nm, either individually or in combination, totaling 10 J/cm^2^. The results revealed that iADMSCs incubated for 7 days in media supplemented with chemical inducers and subjected to PBM exhibited an augmented transdifferentiation process. The addition of PBM primed the cells for differentiation, as indicated by the observed increase in ROS production in the PBM-treated experimental groups. Notably, G PBM demonstrated significant potential for expediting the differentiation process, as evidenced by the reduction in CD44 expression, signifying a faster loss of stem-like properties compared to other groups. These findings underscore the promising utility of investigating PBM to enhance the transdifferentiation of ADMSCs, offering potential therapeutic avenues for neurodegenerative diseases and brain injuries resulting from mechanical trauma.

Moving forward, further exploration into differentiating iADMSCs into neuronal-like cells using PBM should focus on evaluating and documenting the extent of cellular projections or extensions from the main cell body, including quantitatively measuring the length of neurites, which serves as an indicator of neuronal differentiation level. Also, the long-term effects of PBM on the differentiation of iADMSCs into neuronal-like cells should be investigated as it can provide insights into the stability and functionality of the differentiated cells over extended periods, offering a more comprehensive understanding of their therapeutic potential. Additionally, further research could delve into optimizing the parameters of PBM, such as wavelength, fluence, and duration of exposure, to enhance its efficacy in promoting transdifferentiation while minimizing potential adverse effects. Furthermore, the study could benefit from examining the molecular mechanisms underlying the observed effects of PBM on transdifferentiation. Investigating the signaling pathways involved in PBM-induced differentiation could uncover novel targets for modulating cellular fate and optimizing therapeutic outcomes. Moreover, exploring the interplay between PBM and other factors known to influence stem cell differentiation, such as growth factors or extracellular matrix components, could provide valuable insights into synergistic approaches for enhancing neuronal differentiation efficiency.

As for limitations, this study primarily focused on in vitro models, and translating these findings to in vivo settings would be essential for validating their clinical relevance. Additionally, while the study demonstrated the efficacy of PBM in promoting transdifferentiation, its specific effects on neuronal function and maturity warrant further investigation. Addressing these limitations could strengthen the clinical applicability of PBM-based approaches for treating neurodegenerative diseases and brain injuries.
